# Positive psychology in the working environment. Job demands-resources theory, work engagement and burnout: A systematic literature review

**DOI:** 10.3389/fpsyg.2022.1022102

**Published:** 2022-09-20

**Authors:** Michael D. Galanakis, Elli Tsitouri

**Affiliations:** American College of Greece, Athens, Greece

**Keywords:** work engagement, burnout, employee wellbeing, systematic review, job demands-resources theory

## Abstract

The purpose of the present systematic review is to examine the Job Demands-Resources (JD-R) model in order to pinpoint how applicable and relevant is the present theoretical framework in the 21st Century workplace environment. Initially, there will be an examination of the key concepts of the theory, followed by a brief investigation of the empirical validity and importance of the theory in the workplace environment. Then, there will be an empirical investigation of various studies of both cross-sectional and longitudinal nature in the form of a methodology, offering substantial empirical evidence that attests to the validity and effectiveness of the JD-R model in predicting work engagement and burnout-two independent and contrasting states of employee wellbeing, covering the entire spectrum from employee wellness to employee ill-health. We hope this review contributes to the advancement of the JD-R model, aiding researchers and practitioners to obtain a better understanding of the current state of the JD-R model, whilst also offering avenues for future development of the theory, ultimately resulting in a better prediction of employee wellbeing.

## Introduction

The most widely used paradigm in occupational health and in positive psychology at the moment for examining the relationships between job characteristics and employee wellbeing is the Job Demands-Resources (JD-R) model. The model has gained traction from both scholars and practitioners since it was initially published in 2001. According to *Google Scholar*, the three most important papers on the JD-R model (Demerouti et al., 2001; Schaufeli and Bakker, 2004; Bakker and Demerouti, 2007) have received almost 7.000 citations as of January 2015, with the original paper proposed by Demerouti et al. (2001) possessing more than 13.000 citations today. Additionally, since the model's creation about 20 years ago, thousands of organizations have utilized it and the model has sparked hundreds of empirical studies. The JD-R model's hypotheses have also been widely used and generally supported in fields like organizational behavior, occupational psychology, human resource management and a variety of other fields (Bakker and Demerouti, 2016).

Demerouti et al. (2001) developed the JD-R model to better understand burnout, a “*persistent state of work-related stress*” marked by *exhaustion* (feeling emotionally expended and low on energy), *mental distancing* (lack of excitement and cynicism) and *decreased professional efficacy* (doubts about one's skill and ability to contribute at work; Schaufeli et al., 2002, p. 74). After some time, Bakker and Demerouti (2007) proposed a revised version of the JD-R model that included work engagement, a “*positive, fulfilling affective - motivational state of work-related wellbeing*,” defined by *vigor* (high levels of energy and perseverance when faced with difficulties), *dedication* (experiencing a strong sense of fulfillment, inspiration, pride and challenge) and *absorption* (being completely focused and totally immersed in one's work; Bakker et al., 2008, p. 187). As a result, the updated JD-R model sought to examine both a negative and positive psychological state of employee wellbeing-burnout and work engagement-as two opposite poles of a continuum. It is interesting to note, that the JD-R model took 10 years from Demerouti et al. (2001) pioneering work and hundreds of research studies conducted in order to reach its current status as the acclaimed and universally accepted JD-R theory.

Overall, the purpose of the present paper is to offer a synthesis and review of the present literature pertaining to the JD-R model, focusing on current and emerging findings. Furthermore, despite the JD-R model having been explored in relation to a variety of outcomes, the current systematic review only focuses on burnout and work engagement as the original outcome variables of the JD-R model. Additionally, to address the several modifications of the JD-R model, the role of personal resources will also be examined. The function of personal resources in the JD-R model has been the subject of different propositions, according to existing research. We ultimately decided to focus on two theoretical propositions, that we believe provide important avenues for future research; the fact that personal resources mediate the relationship between job resources and wellbeing but also that personal resources can have a direct impact on the wellbeing of individuals. Overall, the hypotheses of the present study were the following:

- Job demands are positively associated to burnout.
- Job resources are positively associated to work engagement.
- Lack of job resources predicts burnout.
- Personal resources mediate the relationship between job resources and wellbeing (work engagement/ burnout).
- Personal resources directly impact wellbeing.

In order to respond to the research objectives outlined above, the remainder of this paper is organized as follows. The next section will extend the presentation of the JD-R model and illustrate its major components and propositions as well as the applicability and importance of the JD-R model in the workplace environment. Afterwards, we will explain comprehensively the systematic review approach that was employed in order to systematically assess the use of the JD-R model in empirical research. The major findings of our rather streamlined systematic review will then be presented, followed by a discussion. The paper concludes with an overview of the key findings, limitations, recommendations for future research and practical implications.

### JD-R model and key propositions

**Figure 1:**
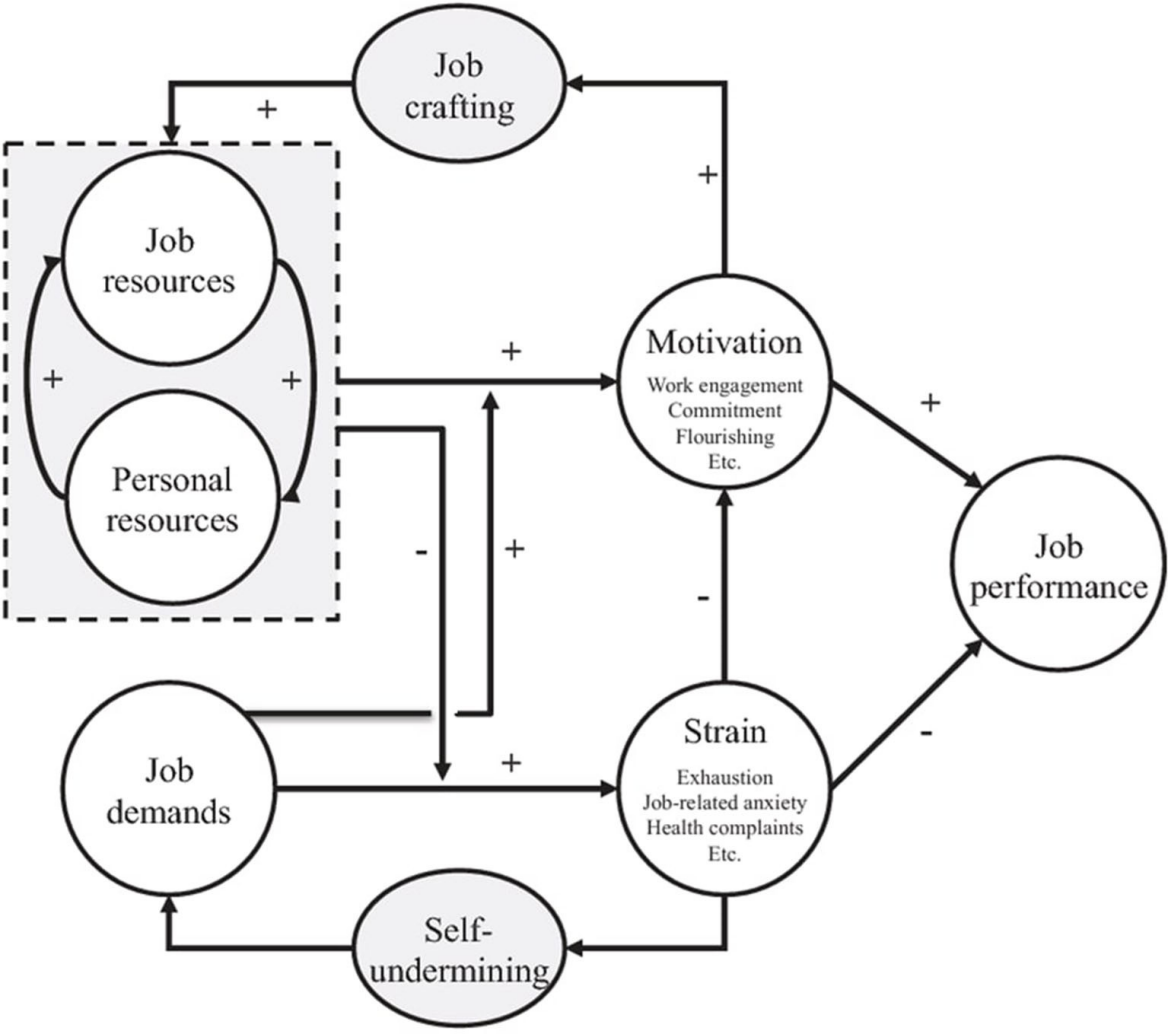
The job demands-resources (JD-R) model, adapted from Bakker and Demerouti (2016).

The JD-R model is a theoretical framework that seeks to combine the stress research approach with the motivation research approach, two relatively independent research paradigms. It is based on well-known stress models like *Karasek's Job Demand Control Model* (1979) and *Siegrist's Effort-Reward Imbalance Model*. The JD-R model's basic premise is that every profession has unique risk factors connected to work-related stress. These factors fall into two broad categories: job demands and job resources, yielding a comprehensive, holistic model that can be applied to a variety of occupational settings, regardless of the specific demands and resources implicated (see Figure 1). According to Demerouti et al. (2001), job demands are defined as “*those physical, social or organizational components of the job that require persistent physical or mental effort and are consequently connected with particular physiological and psychological costs*” (p. 501). Role conflict, time and workload pressure as well as quantitative workload, are the most prevalent examples of job demands. While job resources are considered to be “*those physical, social or organizational aspects of the job that are*: *functional in achieving work goals*; *decrease job demands and the associated physiological and psychological costs and enhance personal growth, learning and development*” (Demerouti et al., 2001, p. 501). Examples of the most prominent job resources are support from others, performance feedback, job control and autonomy among others.

**Figure 2:**
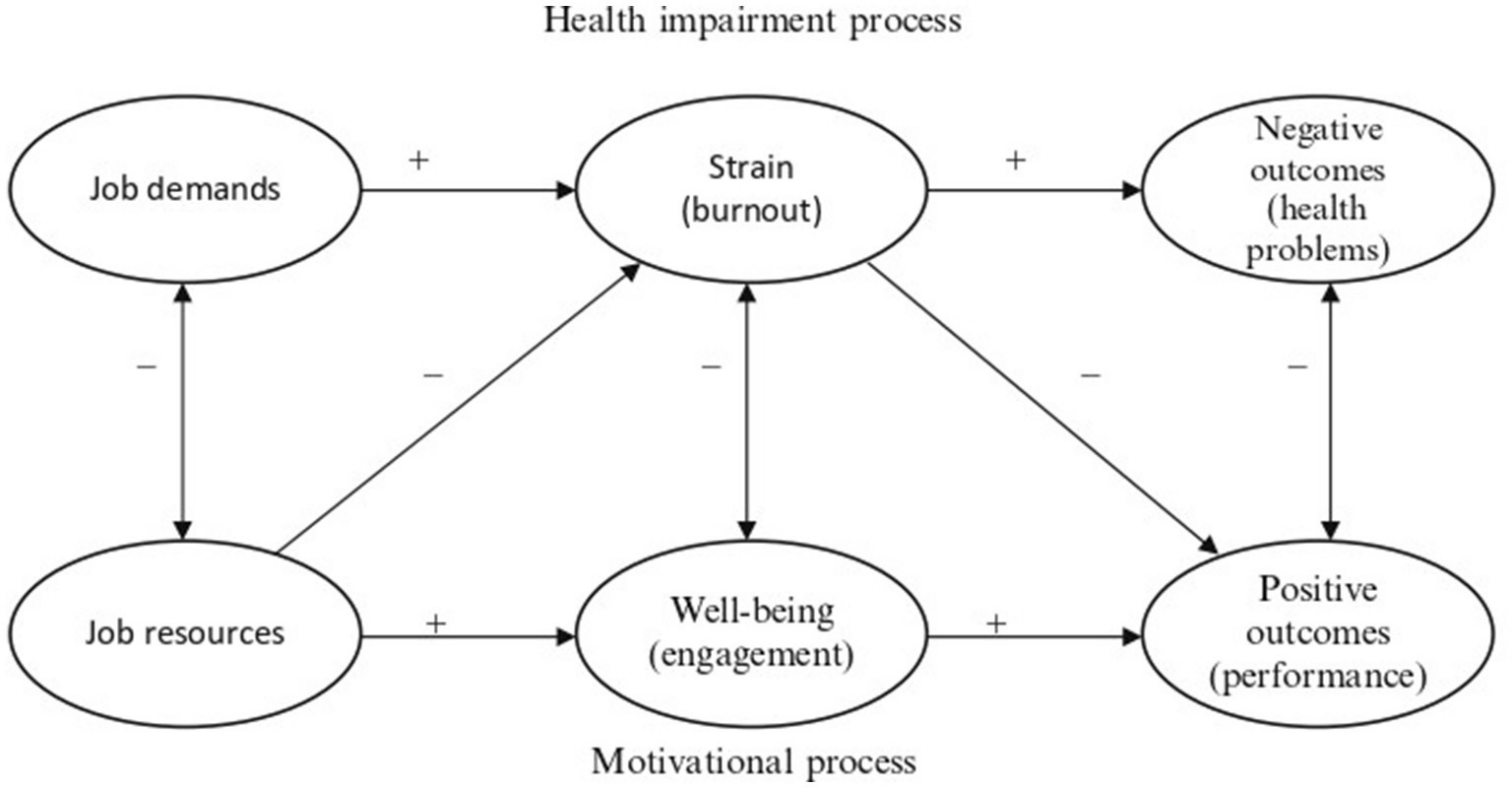
The health-impairment and motivational role of JD-R theory, adapted by Schaufeli and Taris (2014).

The second pillar of the JD-R model is that the emergence of work-related strain and motivation is influenced by two distinct physiological processes. The first is a process of *health impairment*, which contends that demanding jobs or jobs with ongoing demands (such as work overload, emotional demands etc.) drain workers' mental and physical resources and may consequently result in the loss of energy and health issues. Basically, when job demands are consistently high and are not counterbalanced by job resources, employees' energy is gradually depleted resulting in a state of exhaustion (burnout), which may have adverse effects for both the individual (e.g., ill-health) and the organization (e.g., poor performance; Demerouti and Bakker, 2011). The second process put forth by the JD-R model is *motivational* in nature and assumes that job resources have the capacity to motivate and can result in high levels of work engagement, low levels of cynicism and excellent job performance (Hakanen and Roodt, 2010). Accordingly, job resources may play an extrinsic motivational function because they are crucial to attaining work goals or they may play an intrinsic motivational role because they stimulate employees' learning, growth and development (see Figure 2).

The JD-R model also suggests that the interaction between job demands and job resources plays a crucial role in the emergence of job strain and motivation. As such, job resources may reduce the negative effects of job demands on one's health; when faced with high workload expectations, employees may experience less burnout if they have a variety of job resources at their disposal. The JD-R model's final and most recent proposition states that job resources have the greatest impact on motivation or work engagement when job demands are high. In other words, during challenging circumstances, job resources become especially important and salient. Specifically, when a worker is faced with high work demands, job resources become increasingly more valuable and prompt commitment and dedication to current responsibilities (Bakker et al., 2007).

It is also worth noting that an important extension of the JD-R model has been the integration of personal resources. Personal resources are the psychological traits or qualities of the self that are typically linked to resiliency and that allude to the capacity to successfully influence and control one's surroundings (Xanthopoulou et al., 2007). Such favorable self-evaluations can predict motivation, job and life satisfaction, performance at work, among various other desirable outcomes. This is because the more personal resources an individual possesses, the higher their self-esteem and likelihood of experiencing goal self-concordance is Bakker et al. (2014). Self-efficacy, organizational based self-esteem and optimism are considered to be the most prominent examples of personal resources (Xanthopoulou et al., 2007). Specifically, they are regarded as antecedents of job demands and job resources since they facilitate the experience of job resources while also preventing the occurrence of job demands. Furthermore, they moderate the relationship between job characteristics and work outcomes. As a result, high levels of personal resources can reduce the negative impact of high job demands on strain but can also boost the already beneficial effects of high job resources on motivation. Finally, personal resources can also act as mediators between job characteristics and work outcomes; the premise is that job characteristics, particularly job resources, will create personal resources, which in turn will have a direct positive effect on work engagement (Taris and Schaufeli, 2015).

### Empirical validity and importance of the JD-R model in the workplace environment

The empirical support for the JD-R model is robust. The JD-R model has been proven effective across a wide range of occupations, including those requiring high levels of skill (such as those held by teachers, police officers, dentists, managers, nurses) and low levels of skill (such as those held by call center agents, employees of fast-food restaurants, hotel staff as well as students and volunteers). Studies comparing blue- and white-collar workers as well as studies utilizing heterogeneous samples further confirm the model's generalizability (Brough et al., 2013). The model has also been replicated in both qualitative and quantitative designs and it has been found to be quite stable across gender and age. It is also important to note that the JD–R model has been validated in several European countries, such as Finland, the Netherlands, Belgium, Spain, China, Australia and Nigeria. All of these studies certainly provide sufficient evidence of the cross-cultural stability of the model (Van den Broeck et al., 2013; Bakker and Demerouti, 2014, 2018).

One of the most important strengths of the JD-R model is its current flexibility. As previously mentioned, the theory contends that all working environments or job characteristics can be modeled and thus explained by using two different categories, namely job demands and job resources. Accordingly, the JD-R model is not limited to any particular job demands or job resources; it operates under the premise that any demand or resource may have an impact on employee health and wellbeing. Thus, the theory can be adjusted to the particular occupation under consideration and can be applied to all work contexts. For instance, a certain job demand may be important in occupation A but not in occupation B. In the framework of the JD-R model, such diverging conclusions are not inherently problematic since they just represent the fact that not all demands are equally relevant across all job settings. The broad scope of the model appeals to researchers, just as its flexibility is attractive to practitioners (Taris and Schaufeli, 2015). Additionally, the JD-R model differs from earlier models in that rather than connecting clearly defined and specific concepts to one another, the model is heuristic in its form, offering a method of thinking about how work attributes may affect health, wellbeing and motivation. This implies that two studies may be based on and assess the same assumptions of the JD-R model even if there is not any overlap in terms of the concepts being examined. The JD-R model's widespread use in both research and practice is apparently due to its heuristic application as well as due to its broad scope and flexibility (Schaufeli and Taris, 2014).

## Method

A first observation when engaging with JD-R research is that the model has been utilized increasingly by researchers, since its inception in 2001. However, very few studies, including both meta-analyses and systematic reviews, have attempted to examine and evaluate the literature in a standardized manner, examining both burnout and engagement as outcome variables (e.g., Crawford et al., 2010; Bakker et al., 2014; Rattrie and Kittler, 2014; Lesener et al., 2019; Mäkiniemi et al., 2021). In order to provide a systematic and critical evaluation of the current state of the JD-R model in accordance with empirical literature, the present paper will employ a systematic review. Such a selection is justifiable since a well-conducted systematic review will provide us with a thorough, balanced overview of the available research that takes into account various conceptual frameworks while preserving rigor for evaluating the evidence (Thorpe et al., 2005). Therefore, we contend that a systematic methodology is a crucial tool for promoting evidence-based research, giving us a more thorough understanding of the current state of JD-R-related research as well as of potential gaps and avenues for further research. Additionally, there are several fundamental criteria for conductive effective systematic reviews, that the present review attempted to adhere to (e.g., transparency, focus, clarity, synthesis). Given the descriptive nature of the studies under review, the present study adhered to PRISMA standards when feasible. Institutional review board permission was not requested because this was a systematic study that did not include any human subjects.

### Search strategy

An extensive literature search was conducted during June-July 2022. Specifically, a systematic literature search was performed for the period: 2001-2022, given that the primary article of the JD-R model was published in 2001 (Demerouti et al., 2001). Databases utilized included in the search were: *Google Scholar, PsycInfo, PsycArticles* and *Academic Search Complete (EBSCO)*. Additional articles were found by scanning the reference lists of such articles, as well as by conducting manual searches of pertinent books. All articles were examined based on title and abstract and labeled as “*eligible*” or “*ineligible*.” The search included the terms: *burnout, work (or employee) engagement, job demands, job resources, personal resources* and *JD-R model*. Specifically, keywords relevant for engagement were: *vigor, dedication, absorption* while for burnout relevant keywords were: *exhaustion, cynicism, inefficacy* and *depersonalization*. Furthermore, two additional search terms were added: *cross-sectional, longitudinal*, in order to further filter results with a specific research design, given that we wanted to obtain a general overview of both cross-sectional and longitudinal research on the JD-R model.

The final search phrase used in queries was: (“work engagement” or “employee engagement” or “vigor” or “dedication” or “absorption”) AND (“burnout” or “cynicism” or “exhaustion” or “inefficacy” or “depersonalization”) AND (“job demand”) OR (“job resource') OR (“personal resource”) AND (“job demands-resources model” or “JD-R model”) AND (“cross-sectional” or “longitudinal”).

### Inclusion criteria

The inclusion criteria for studies were the following:

- *Type of Study:* Studies should focus on connecting work engagement or burnout to the JD-R model. Additionally, studies ought to measure at least one job characteristic (demand or resource).
- *Participants:* Studies should utilize employees as subjects of their research, in accordance with the work-oriented nature of the JD-R model. In addition, for the results to be statistically significant, the sample size for such investigations should consist of at least 100 individuals.
- *Study Design:* Studies should be of empirical nature. Specifically, studies should be of cross-sectional or longitudinal design.
- *Publication Status:* Included studies should be published in English, peer-reviewed scientific journals.
- *Year of Publication:* Studies should have been published from 2001 and after, given that in 2001 the foundational article of the JD-R model was published.

## Results

Twenty articles were included in the present review and assessed in accordance with our research objectives. The study sample sizes ranged from 146 to 11.468, nearly all utilizing a mixed gender sample. There were a range of occupations, with no industry or job type receiving more emphasis than others. The participants in the studies of our sample were drawn from a range of 12 different countries. In total, 5 studies used participants from Finland (23%), 3 studies used participants from China (14%), 2 studies used participants from Netherlands (9%), 2 studies used participants from South Africa (9%) and 2 studies utilized participants from Australia (9%). The rest of the studies utilized participants from different countries of origin, i.e., Austria, Belgium, Africa, Italy, Spain, Sweden and Switzerland. Evidently, there is a European bias in regard to the nationality of participants and country of origin of the institutions where researchers were employed, with the majority of JD-R related research being carried out in European countries. Furthermore, the majority of the studies utilized a cross-sectional design, specifically 11 studies utilized a cross-sectional design (23%), 7 studies utilized a longitudinal design (15%) and 2 studies utilized a mixed methods design (4%), otherwise known as a cross-sequential design (e.g., utilizing both a cross-sectional and longitudinal data set; see Table 1). For longitudinal studies, duration ranged from 8 months to 7 years, with majority of studies (82%) utilizing a two-wave approach, with only 2 studies (9%) employing a three-wave approach. In regard to the method of analysis, 11 studies utilized structural equation modeling (50%), 5 studies utilized regression analysis (23%), two studies utilized discriminant analysis (9%) and another 2 studies utilized principal component analysis (9%).

**Table 1:** Overview of the identified studies of the JD-R model.

**Primary study**	**Country**	**Participants (N)**	**Study design**	**Method of analysis**
Bakker et al. (2004)	Netherlands	146	Cross-sectional	Structural equation modeling
Bakker et al. (2007)	Finland	805	Cross-sectional	Hierarchical regression analysis
Brauchli et al. (2013)	Switzerland	3.045	Longitudinal	Structural equation modeling
Brough et al. (2013)	Australia and China	9.404	Cross-sequential	Hierarchical multiple regression analysis
Consiglio et al. (2013)	Italy	5.407	Cross-sectional	Multilevel structural equation modeling
De Beer et al. (2013)	South Africa	593	Longitudinal	Structural equation modeling
Hakanen et al. (2005)	Finland	3.255	Cross-sectional	Hierachical regression analysis
Hakanen et al. (2008)	Finland	3.035	Longitudinal	Structural equation modeling
Hakanen et al. (2021)	Finland	11.468 (cross-sectional data set), 2.334 (longitudinal two-wave data set)	Cross-sequential	Discriminant analysis
Hu et al. (2017)	China	445	Longitudinal	Principal component analysis
Korunka et al. (2009)	Austria	956	Cross-sectional	Structural equation modeling
Kotze (2018)	Africa	407	Cross-sectional	Structural equation modeling
Lorente Prieto et al. (2008)	Spain	274	Longitudinal	Hierarchical multiple regression analysis
Patience et al. (2020)	South Africa	420	Cross-sectional	Regression analysis
Peterson et al. (2008)	Sweden	3.719	Cross-sectional	Linear discriminant analysis
Salmela-Aro and Upadyaya (2018)	Finland	1.415	Cross-sectional	Structural equation modeling
Schaufeli et al. (2009)	Netherlands	201	Longitudinal	Structural equation modeling
Van den Broeck et al. (2017)	Belgium	2.585	Cross-sectional	Structural equation modeling
Vinod Nair et al. (2020)	Australia	171	Cross-sectional	Structural equation modeling
Wang et al. (2016)	China	263	Longitudinal	Principal component analysis

Furthermore, out of 20 studies, 11 studies (50%) examined both burnout and work engagement, 5 studies (23%) examined only work engagement and 4 studies (18%) examined only burnout. Out of these studies, 16 studies (73%) measured burnout through the *Maslach Burnout Inventory* (Maslach and Jackson, 1986), followed to a lesser extent by three studies (14%) utilizing the *Oldenburg Burnout Inventory* (Halbesleben and Demerouti, 2005; Demerouti and Bakker, 2008) and one study (4.5%) utilized the *Burgen Burnout Inventory* (Salmela-Aro et al., 2011). As for work engagement, all studies utilized the *Utrecht Work Engagement Scale* (Schaufeli et al., 2002). Additionally, 14 studies (67%) examined both job demands and resources, five studies (23%) examined only job resources and 1 study (4.5%) examined only job demands; in that extent, four of these studies (18%) examined the role of personal resources. Out of the four studies that investigated personal resources, three of these studies (14%) found evidence that personal resources mediate the relationship between job resources and engagement/ burnout, with two of the studies also suggesting that they directly impact wellbeing.

The most prevalent job demands were: work overload (quantitative/qualitative), emotional demands and role conflict/ambiguity, followed closely by the physical work environment and home-family conflict. The most prominent job resources were: support (either from supervisors or co-workers), autonomy, job control, opportunities for learning and development, performance feedback and role clarity. While, for personal resources, the studies that examined personal resources are too limited in order to investigate potential patterns; nevertheless, in the present studies, the personal resources examined were: psychological capital (PsyCap), self-efficacy, optimism, mental and emotional competencies and self-esteem.

Moreover, the majority of studies (82%) found conclusive support both for the motivational and health-impairment processes of the JD-R model (18 out of 20 studies), with only two studies (9%) finding support only for the motivational pathway. Specifically, the reviewed studies found robust evidence that increased job demands and lack of resources contribute to burnout, while also having found that job resources positively predict work engagement. In regard to the coping hypothesis of the JD-R model, three studies assessed whether job resources become particularly salient in the presence of high job demands, with all studies having found concrete evidence. As for the proposition of the JD-R model regarding reciprocal causation, the number of studies (two) assessing the reversed pathways of the JD-R model are too limited in order to allow for any patterns or further explanations to be identified. Four studies (18%) also found support for the presence of loss/ gain spirals, in accordance with Hobfoll's *Conservation of Resources (COR) Theory*. Finally, only two studies (9%) investigated the differentiation between *challenge* and *hindrance demands*, with both studies finding evidence that challenge demands can have a beneficial role in promoting the engagement and motivation of employees.

## Discussion

The present systematic literature review consisted of 20 studies which met the eligibility criteria and could thus be included. This study aimed to address four hypotheses, in accordance with the JD-R model: (a) job demands are positively associated to burnout; (b) job resources are positively associated to work engagement; (c) lack of job resources predicts burnout, (d) personal resources mediate the relationship between job resources and wellbeing (work engagement/ burnout) and (e) personal resources directly impact wellbeing. As can be seen, nearly all of the effects received support (albeit at different levels), suggesting that the JD-R model can act as a valuable tool for predicting burnout and work engagement. Almost all of the studies examined, confirmed the dual pathway to employee-wellbeing, namely that job demands and resources are the catalysts of two relatively distinct processes, namely a health impairment process and a motivational process. Consequently, job demands are typically the most significant predictors of outcomes like burnout, psychological strain and exhaustion, while job resources are typically the most important determinants of motivation and work engagement. These findings corroborate JD-R theory's assertion that job demands and job resources elicit two unique psychological processes that ultimately have an impact on significant organizational outcomes (Schaufeli and Taris, 2014).

Additionally, our hypothesis that a lack of job resources predicts burnout was further validated, confirming Maslach et al. (2001) proposition that a lack of job resources will instill a self-protective process, resulting in diminished motivation and disengagement from work (e.g., the motivational component of burnout). In that respect, our systematic review also confirmed the fourth proposition of the JD-R model, namely that job resources are particularly motivating when job demands are high. Consistent with previous research, job resources are most conducive to sustaining work engagement under conditions of high job demands; in other words, job resources become more salient and acquire their motivational potential when employees are faced with high job demands (Bakker and Demerouti, 2007). Additionally, although limited, from the studies included, it was found that the differentiation of *challenge* and *hindrance demands* is crucial, with evidence pointing out to the fact that while challenge demands may be linked to strain variables, they can also be advantageous because they can enhance employees' motivation and work engagement. Consistent with LePine et al. (2005) claim, challenge demands can be potentially gratifying work experiences that can lead to opportunities for personal development, stimulation and success.

As for personal resources, the studies are too limited to draw any concrete conclusions; nevertheless, the findings of the reviewed studies demonstrated that personal resources can influence employees' wellbeing, particularly they can buffer the unfavorable effect of job demands, thereby reducing burnout as well as enhance employees' work engagement, given that they increase employees' belief about being able to adequately fulfill their tasks and achieve desired outcomes at work. This is consistent with Xanthopoulou et al. (2007) claim that personal resources can enhance employees' resiliency and perceived ability and that by enabling successful control of their environment, personal resources can help employees to achieve positive health outcomes in the future. As for our hypothesis that personal resources mediate the relationship between job resources and wellbeing (work engagement/burnout), results were mixed with some studies (both cross-sectional and longitudinal) confirming their mediating role, while others failing to confirm their mediating effect. Clearly, more research is needed in order to fully comprehend the function of personal resources within the JD-R model.

Moreover, evidence of reversed causation and of gain and loss cycles was found by some studies. Based on JD-R theory, within positive gain cycles, job resources stimulate work engagement which, in turn, increases job resources, prompting further engagement. While, loss cycles refer to processes in which burnout induces resource loss and an accumulation of job demands, which contribute to further burnout. These findings indicate that work engagement may enable the deployment of job resources. As such, engaged employees, who are intrinsically driven to complete their objectives at work, will activate or create job resources to utilize as a means of fulfilling these objectives. While, employees experiencing strain or disengagement often behave in a way that places additional demands upon them, making it harder for them to cope with job demands in an effective manner and thus initiating the subsequent loss of job resources and an increase in job demands (Xanthopoulou et al., 2009).

### Limitations

Despite our best attempts to provide a thoroughly conducted systematic review that adheres to acceptable standards, our study possesses certain limitations. The key limitation of the present review is that not all existing studies were taken into account. This is a frequent shortcoming in systematic reviews. The searches were limited to titles, abstracts and keywords, so it is plausible that some pertinent studies were missed. In this regard, we also concentrated on databases that are dedicated to psychology, such as *PsycInfo* and *PsycArticles*. However, in order to address this limitation and to cover a wider spectrum of studies, we also included multidisciplinary databases, such as *Academic Search Complete* and *Google Scholar*. Certainly, it is possible that we did not discover every study that employed the JD-R model as its conceptual framework. Other databases that were not taken into consideration may reveal additional studies that were not included in the present review. For instance, it is likely that possible that relevant studies may have been published in business and management oriented journals, which were excluded from our database search. Furthermore, papers published in languages other than English were not included; this should certainly be taken into account when interpreting the findings. The low sample size of the studies included in the present systematic review should also be taken into consideration, given that it undoubtedly limited the extent to which inferences can be formed. However, it is important to note that, in organizational psychology research, small numbers are pervasive (Knight et al., 2017).

Furthermore, we concentrated on peer-reviewed, published literature due to the vast amount of relevant studies which was sufficient in order to examine our research objectives as well as due to the higher caliber and rigor of such research. This might have contributed to publication bias (file-drawer effect). However, research indicates that such a bias is unlikely and does not seriously jeopardize the validity of the study (Dalton et al., 2012; Van Aert et al., 2019). To minimize the occurrence of such bias, we did not concentrate on effect sizes or significance levels in the studies reviewed, but rather on general approaches and conceptualizations of studies, relating work engagement and burnout to the JD-R model. Finally, all of the studies relied solely on the UWES, in order to measure work engagement. This does not accurately depict the rather fragmented nature of the field in regard to the measurement of work engagement, but it does reflect the prevalence of the measure. Its dominance, however, does not make it the “*best*” measure and its validity and reliability have most recently come into question, given that the UWES overlaps with a number of job attitudes including stress, organizational commitment, job performance and burnout (Byrne et al., 2016). At the same time, however, it could also be considered as an advantage given that results obtained from utilization of the same scale are standardized, making comparisons easier and more meaningful. As such, we were able to more effectively synthesize and understand the present findings, which would have been considerably more challenging if numerous different work engagement measures and definitions had been examined simultaneously.

### Future research directions

Although the JD-R model possesses substantial empirical validity as proven by the systematic literature review conducted, there are several unanswered questions which ought to be further investigated in future research. The synthesis of JD-R related research allows us to make judgements and reach certain conclusions “*about what we currently know and do not know”* in regard to the current state of the JD-R model (Rattrie and Kittler, 2014, p. 271). The most important ones will be emphasized in the present study. The JD-R model makes the fundamental assumption that most (if not all) job characteristics can be seamlessly separated into two broad categories: demands and resources, respectively. As such, one avenue for future research would be to look into the nature of demands and resources. The conceptual gap between job demands and job resources is not as clear-cut as it might initially seem. On the one hand, a shortage of a certain job resource may be seen as a demand; for instance, a lack of resources at work indicates that employees must put in more effort to meet their objectives at work. This implies that a scarcity in job resources is equivalent to an excess of job demands.

On the other hand, not all job demands in the JD-R model seem to be equal. Based on the conceptual distinction between *challenge demands* and *hindrance demands*, Van den Broeck et al. (2010) demonstrated that high levels of hindrance demands- threatening demands that hinder employees' control and cannot easily be conquered- were associated with lower vigor and higher exhaustion at work, whereas challenging demands- demands that not only require effort in order to effectively address them but are also stimulating in their own right and aid in the achievement of work objectives—were related to positive outcomes, such as higher vigor. The meta-analysis by Crawford et al. (2010) substantiated these results. It appears that the distinction between job demands and resources in the early versions of the JD-R model is not as straightforward as first believed. Thus, future studies should investigate this subject, preferably across a variety of job types and different types of demands (and possibly resources; Demerouti and Bakker, 2011). They should also attempt to identify the circumstances in which job demands serve as hindrances vs. challenges (Podsakoff et al., 2007; Schaufeli and Taris, 2014).

Moreover, the JD-R model proposes clear-cut, one-directional causal relations between demands, resources and outcomes. Numerous empirical studies have validated the model's central propositions, which contend that job demands predict job strain and job resources predict motivation. However, it is also feasible that job demands and resources are influenced by employee wellbeing. Although research has frequently established that longitudinal reciprocal effects exist between job demands and burnout as well as with job resources and work engagement, it is not clearly understood how a gain (or loss) spiral would manifest itself or even if such a spiral truly exists. As such, given that assuming linear causality is unnecessarily simplistic, future research should examine more methodically the complicated relations among the concepts in the model, in order to obtain to better comprehend the reciprocal relationships among key variables in the model (Taris and Schaufeli, 2015; Lesener et al., 2019). Evidently, more well-designed and thoroughly investigated research on this intriguing subject is required.

The role of personal resources should also be further investigated; the JD-R model can incorporate personal resources in a variety of ways. Currently, there is no single optimum approach for broadening the JD-R model in order to allow for the inclusion of personal resources into the model. For instance, they can be integrated as mediators, moderators, antecedents of job demands and job resources or as any combination of these. Future research should systematically examine the various roles of personal resources, “*comparing different conceptualizations of the relations between personal and job resources, job demands and outcomes*” (Schaufeli and Taris, 2014, p. 51). Future studies should examine the possibility of a three-way interaction between job demands, job resources and personal resources, that is a by-product of the complicated relationship between personal resources and the workplace environment (Taris and Schaufeli, 2015). This would imply a two-way interaction of personal resources with job demands and job resources, respectively.

Finally, future studies should utilize objective indicators at a greater extent. Most JD-R related research has relied on self-report measures. The issue with such measures is that since one individual provides all the data, common method bias may cause statistical relations amongst constructs to be overestimated. Thus, it is vital for the advancement of the field of organizational psychology to include in research studies, objective metrics that have a direct impact in business. Therefore, future studies should build on earlier work by utilizing longitudinal designs, which provide more credible conclusions to be reached in regard to the causal pathways of the JD-R model. Future research should further clarify the extent to which the JD-R model predicts objective business metrics (i.e., sales, customer satisfaction, work performance, sickness absenteeism etc.). In that respect, the ability to predict objective health outcomes, whilst using both job demands and resources as key predictors would also be worthwhile to investigate (Bakker and Demerouti, 2007, 2016).

### Practical implications

Organizations can utilize the JD-R model as a framework in order to enhance employee wellbeing and motivation while also optimizing various organizational outcomes. First of all, the JD-R model does not restrict the study concepts that can be potentially investigated. It is therefore theoretically applicable to a very broad range of job and personal characteristics and outcomes, as opposed to concentrating on a narrow range of factors that are supposed to account for a rather limited set of outcomes (Bakker and Demerouti, 2016). Practically speaking, this suggests that the model may be modified to accommodate the specific requirements of a company in any situation. This greatly enhances the model's applicability in a multitude of settings. The JD-R model accordingly presupposes that while each occupation may have distinct characteristics, these characteristics can be grouped into two general categories (i.e., job demands and job resources), constituting an all-encompassing model that may be applied to numerous occupational settings, regardless of the specific demands and resources implicated. Given the complexity of today's work, identifying the demands and resources that may be harmful to health or deplete motivation is a crucial first step toward enhancing wellbeing in the workplace (Bakker and Demerouti, 2007). In that regard, the JD-R model also appeals to various occupational groups responsible for the management of an organization's human resources. As such, human resources experts are drawn to the “*positive*” motivational perspective whereas occupational health professionals are drawn to the “*negative*” stress perspective. The JD-R approach might therefore can potentially bridge the divide between occupational health management and human resources management. From the standpoint of the JD-R model, these two viewpoints are not only equally acceptable, but they are also intertwined with one another (Schaufeli and Taris, 2014).

The JD-R model has also been found to be of essence in organizational practice since it assists practitioners and organizations in identifying the elements necessary for optimal wellbeing (such as motivation and health) as well as for effective performance in the workplace. In that respect, organizational assessment is a an especially important practical application. Most organizations interested in employee wellbeing want to determine the extent of potential job demands and job resources. Essential job demands and resources are measured at the individual level in an organizational assessment and overall organizational scores are compared to industry/national benchmarks. Additionally, the organizational report contains crucial data about job demands, resources, wellbeing as well as the performance of various teams, departments etc. (Bakker and Demerouti, 2018). This intervention is typically created through discussion between managers and employees, who brainstorm in workshops about potential solutions for suboptimal work environments.

Finally, job redesign and job crafting interventions are also two additional significant practical implications of the JD-R model. Job redesign is a structural organizational intervention that seeks to modify the determinants of employee wellbeing— namely their job demands and resources. Specifically, it refers to the process through which an organization or supervisor modifies a job, its tasks or an employee's working environment. As such, the organization or the employees themselves can redesign the structure and content of the work, with the ultimate goal of improving outcomes like employee wellbeing, work engagement and job performance (Schaufeli, 2017). Finally, job crafting interventions are also of essence; job crafting is an individual-level intervention that is usually carried out by the individual worker. By choosing specific tasks, negotiating different job content and giving their tasks or jobs meaning and significance, employees can actively change the design of their jobs (Bakker and Demerouti, 2014, 2016). From a JD-R standpoint, employees can dynamically modify their own job demands and job resources, ultimately resulting in increased engagement, motivation, job satisfaction and thriving at work. Furthermore, by demonstrating to employees how to craft their jobs, organizations can encourage behavior that is advantageous both to their employees and the organization itself (Van Wingerden and Van der Vaart, 2019).

## Conclusion

Overall, the present study provided a comprehensive and systematic review of research on the JD-R model, offering substantial evidence of its applicability and implementation in the workplace environment. It also proved its effectiveness in predicting work engagement and burnout, two especially important forms of employee wellbeing or lack thereof. Since its inception, JD-R theory has inspired hundreds of studies, with its empirical validity attested and supported in numerous studies and organizational settings. However, it is important to bear in mind that the present theoretical framework also possesses certain limitations and unresolved issues, which ought to be further examined by future research in order to develop an even more nuanced understanding of the theory. Nevertheless, despite existing research limitations, the JD-R model offers a truly effective conceptual framework for describing employee wellbeing in a wide range of organizations and occupational domains. This is further proven by the fact that our review corroborated the fundamental propositions of the JD-R model. Various practical applications and interventions in the organizational context have also been developed on the basis of the theory, with the most important ones being discussed. We hope this review contributes to the advancement of the JD-R model, aiding researchers and practitioners to obtain a better understanding of the current state of the JD-R model, whilst also offering avenues for future development of the theory, ultimately resulting in a better prediction of employee wellbeing.

## Data availability statement

The original contributions presented in the study are included in the article/supplementary material, further inquiries can be directed to the corresponding author/s.

## Author contributions

Both authors listed have made a substantial, direct, and intellectual contribution to the work and approved it for publication.

## Conflict of interest

The authors declare that the research was conducted in the absence of any commercial or financial relationships that could be construed as a potential conflict of interest.

## Publisher's note

All claims expressed in this article are solely those of the authors and do not necessarily represent those of their affiliated organizations, or those of the publisher, the editors and the reviewers. Any product that may be evaluated in this article, or claim that may be made by its manufacturer, is not guaranteed or endorsed by the publisher.

## References

[B1] Bakker A. B. Demerouti E. (2007). The job demands-resources model: State of the art. J. Manag. Psychol. 22, 309–328. 10.1108/0268394071073311531861812

[B2] Bakker A. B. Demerouti E. (2014). Job demands—Resources theory, in: Work and Wellbeing: A Complete Reference Guide, eds Chen P. Y. Cooper C. L. (New York, NY: John Wiley & Sons). 10.1002/9781118539415.wbwell019

[B3] Bakker A. B. Demerouti E. (2016). Job demands–resources theory: Taking stock and looking forward. J. Occup. Health Psychol. 22, 273–285. 10.1037/ocp0000056 27732008

[B4] Bakker A. B. Demerouti E. (2018). Multiple levels in job demands-resources theory: Implications for employee well-being and performance, in: Handbook of Well- Being, eds Diener E. Oishi S. Tay L. (Salt Lake City, UT: DEF Publishers).

[B5] Bakker A. B. Demerouti E. Sanz-Vergel A. I. (2014). Burnout and work engagement: The JD–R approach. Annu. Rev. Organ. Psychol. Organ. Behav. 1, 389–411. 10.1146/annurev-orgpsych-031413-091235

[B6] Bakker A. B. Demerouti E. Verbeke W. (2004). Using the job demands-resources model to predict burnout and performance. Hum. Resour. Manage. 43, 83–104. 10.1002/hrm.20004

[B7] Bakker A. B. Hakanen J. J. Demerouti E. Xanthopoulou D. (2007). Job resources boost work engagement, particularly when job demands are high. J. Educ. Psychol. 99, 274–284. 10.1037/0022-0663.99.2.27428150993

[B8] Bakker A. B. Schaufeli W. B. Leiter M. P. Taris T. W. (2008). Work engagement: An emerging concept in occupational health psychology. Work Stress 22, 187–200. 10.1080/02678370802393649 20103890

[B9] Brauchli R. Schaufeli W. B. Jenny G. J. Füllemann D. Bauer G. F. (2013). Disentangling stability and change in job resources, job demands, and employee well-being—A three-wave study on the Job-Demands Resources model. J. Vocat. Behav. 83, 117–129. 10.1016/j.jvb.2013.03.003

[B10] Brough P. Timms C. Siu O. Kalliath T. O'Driscoll M. P. Sit C. H. . (2013). Validation of the Job Demands-Resources model in cross-national samples: Cross-sectional and longitudinal predictions of psychological strain and work engagement. Human Relations 66, 1311–1335. 10.1177/0018726712472915

[B11] Byrne Z. S. Peters J. M. Weston J. W. (2016). The struggle with employee engagement: Measures and construct clarification using five samples. J. Appl. Psychol. 101, 1201. 10.1037/apl0000124 27281184

[B12] Consiglio C. Borgogni L. Alessandri G. Schaufeli W. B. (2013). Does self-efficacy matter for burnout and sickness absenteeism? The mediating role of demands and resources at the individual and team levels. Work Stress 27, 22–42. 10.1080/02678373.2013.769325

[B13] Crawford E. R. LePine J. A. Rich B. L. (2010). Linking job demands and resources to employee engagement and burnout: A theoretical extension and meta-analytic test. J. Appl. Psychol. 95, 834–848. 10.1037/a0019364 20836586

[B14] Dalton D. R. Aguinis H. Dalton C. M. Bosco F. A. Pierce C. A. (2012). Revisiting the file drawer problem in meta-analysis: An assessment of published and nonpublished correlation matrices. Pers. Psychol. 65, 221–249. 10.1111/j.1744-6570.2012.01243.x

[B15] De Beer L. T. Pienaar J. Rothmann Jr S. (2013). Investigating the reversed causality of engagement and burnout in job demands-resources theory. SA J. Indus. Psychol. 39, 1–9. 10.4102/sajip.v39i1.1055

[B16] Demerouti E. Bakker A. B. (2008). The Oldenburg Burnout Inventory: A good alternative to measure burnout and engagement, in: Stress and Burnout in Health Care, ed Halbesleben J. (Hauppage, NY: Nova Sciences).

[B17] Demerouti E. Bakker A. B. (2011). The job demands-resources model: Challenges for future research. SA J. Indus. Psychol. 37, 1–9. 10.4102/sajip.v37i2.974

[B18] Demerouti E. Bakker A. B. Nachreiner F. Schaufeli W. B. (2001). The job demands-resources model of burnout. J. Appl. Psychol. 86, 499–512. 10.1037/0021-9010.86.3.499 11419809

[B19] Hakanen J. J. Bakker A. B. Demerouti E. (2005). How dentists cope with their job demands and stay engaged: The moderating role of job resources. Eur. J. Oral Sci. 113, 479–487. 10.1111/j.1600-0722.2005.00250.x 16324137

[B20] Hakanen J. J. Bakker A. B. Turunen J. (2021). The relative importance of various job resources for work engagement: A concurrent and follow-up dominance analysis. BRQ Business Res. Q. 95, 834–848. 10.1177/23409444211012419

[B21] Hakanen J. J. Roodt G. (2010). Using the job demands-resources model to predict engagement: Analysing a conceptual model, in: Work Engagement: A Handbook of Essential Theory and Research, eds Bakker A. B. Leiter M. P. (New York, NY: Psychology Press).

[B22] Hakanen J. J. Schaufeli W. B. Ahola K. (2008). The Job Demands-Resources model: A three-year cross-lagged study of burnout, depression, commitment, and work engagement. Work Stress 22, 224–241. 10.1080/02678370802379432

[B23] Halbesleben J. R. B. Demerouti E. (2005). The construct validity of an alternative measure of burnout: Investigating the English translation of the Oldenburg Burnout Inventory. Work Stress 19, 208–220. 10.1080/02678370500340728

[B24] Hu Q. Schaufeli W. B. Taris T. W. (2017). How are changes in exposure to job demands and job resources related to burnout and engagement? A longitudinal study among Chinese nurses and police officers. Stress Health 33, 631–644. 10.1002/smi.2750 28371227

[B25] Knight C. Patterson M. Dawson J. (2017). Building work engagement: A systematic review and meta-analysis investigating the effectiveness of work engagement interventions. J. Organ. Behav. 38, 792–812. 10.1002/job.2167 28781428 PMC5516176

[B26] Korunka C. Kubicek B. Schaufeli W. B. Hoonakker P. (2009). Work engagement and burnout: Testing the robustness of the Job Demands-Resources model. J. Posit. Psychol. 4, 243–255. 10.1080/17439760902879976

[B27] Kotze M. (2018). How job resources and personal resources influence work engagement and burnout. Afr. J. Econ. Manage. Stud. 9, 148–164. 10.1108/AJEMS-05-2017-0096

[B28] LePine J. A. Podsakoff N. P. LePine M. A. (2005). A meta-analytic test of the challenge stressor-hindrance stressor framework: An explanation for inconsistent relationships among stressors and performance. Acad. Manage. J. 48, 764–775. 10.5465/amj.2005.18803921

[B29] Lesener T. Gusy B. Wolter C. (2019). The job demands-resources model: A meta-analytic review of longitudinal studies. Work Stress 33, 76–103. 10.1080/02678373.2018.1529065

[B30] Lorente Prieto L. Salanova Soria M. Martínez Martínez I. Schaufeli W. (2008). Extension of the Job Demands-Resources model in the prediction of burnout and engagement among teachers over time. Psicothema 20, 354–360. 18674427

[B31] Mäkiniemi J. P. Ahola S. Nuutinen S. Laitinen J. Oksanen T. (2021). Factors associated with job burnout, job satisfaction and work engagement among entrepreneurs. A systematic qualitative review. J. Small Business Entrepreneurship 33, 219–247. 10.1080/08276331.2020.1764735

[B32] Maslach C. Jackson S. E. (1986). Maslach Burnout Inventory Manual (2nd ed.). Palo Alto, CA: Consulting Psychologists Press.

[B33] Maslach C. Schaufeli W. B. Leiter M. P. (2001). Job burnout. Annu. Rev. Psychol. 52, 397–422. 10.1146/annurev.psych.52.1.397 11148311

[B34] Patience M. G. De Braine R. Dhanpat N. (2020). Job demands, job resources, and work engagement among South African nurses. J. Psychol. Afr. 30, 408–416. 10.1080/14330237.2020.1821315

[B35] Peterson U. Demerouti E. Bergström G. Samuelsson M. Åsberg M. Nygren Å. (2008). Burnout and physical and mental health among Swedish healthcare workers. J. Adv. Nurs. 62, 84–95. 10.1111/j.1365-2648.2007.04580.x 18352967

[B36] Podsakoff N. P. LePine J. A. LePine M. A. (2007). Differential challenge stressor-hindrance stressor relationships with job attitudes, turnover intentions, turnover, and withdrawal behavior: A meta-analysis. J. Appl. Psychol. 92, 438–454. 10.1037/0021-9010.92.2.438 17371090

[B37] Rattrie L. T. Kittler M. G. (2014). The job demands-resources model and the international work context–a systematic review. J. Global Mobility 2, 260–279. 10.1108/JGM-06-2014-001830925279

[B38] Salmela-Aro K. Rantanen J. Hyvönen K. Tilleman K. Feldt T. (2011). Bergen Burnout Inventory: reliability and validity among Finnish and Estonian managers. Int. Arch. Occup. Environ. Health 84, 635–645. 10.1007/s00420-010-0594-3 21082191

[B39] Salmela-Aro K. Upadyaya K. (2018). Role of demands-resources in work engagement and burnout in different career stages. J. Vocat. Behav. 108, 190–200. 10.1016/j.jvb.2018.08.002

[B40] Schaufeli W. B. (2017). Applying the Job Demands-Resources model: A ‘how to' guide to measuring and tackling work engagement and burnout. Organ. Dyn. 46, 120–132. 10.1016/j.orgdyn.2017.04.008

[B41] Schaufeli W. B. Bakker A. B. (2004). Job demands, job resources, and their relationship with burnout and engagement: A multi-sample study. J. Organ. Behav. 25, 293–315. 10.1002/job.248

[B42] Schaufeli W. B. Bakker A. B. Van Rhenen W. (2009). How changes in job demands and resources predict burnout, work engagement and sickness absenteeism. J. Organ. Behav. 30, 893–917. 10.1002/job.595

[B43] Schaufeli W. B. Salanova M. González-Rom,á V. Bakker A. B. (2002). The measurement of engagement and burnout: A two sample confirmatory factor analytic approach. J. Happiness Stud. 3, 71–92. 10.1023/A:1015630930326

[B44] Schaufeli W. B. Taris T. W. (2014). A critical review of the job demands-resources model: Implications for improving work and health, in: Bridging Occupational, Organizational and Public Health: A Transdisciplinary Approach, eds Bauer G. F. Hämmig O. (Dordrecht: Springer). 10.1007/978-94-007-5640-3_4

[B45] Taris T. W. Schaufeli W. B. (2015). The job demands-resources model, in: The Wiley Blackwell Handbook of the Psychology of Occupational Safety and Workplace Health, eds Clarke S. Probst T. M. Guldenmund F. Passmore J. (Chichester: John Wiley & Sons). 10.1002/9781118979013.ch8

[B46] Thorpe R. Holt R. Macpherson A. Pittaway L. (2005). Using knowledge within small and medium-sized firms: A systematic review of the evidence. Int. J. Manag. Rev. 7, 257–281. 10.1111/j.1468-2370.2005.00116.x

[B47] Van Aert R. C. Wicherts J. M. Van Assen M. A. (2019). Publication bias examined in meta-analyses from psychology and medicine: A meta-meta-analysis. PLoS ONE 14, e0215052. 10.1371/journal.pone.0215052 30978228 PMC6461282

[B48] Van den Broeck A. De Cuyper N. De Witte H. Vansteenkiste M. (2010). Not all job demands are equal: Differentiating job hindrances and job challenges in the Job Demands-Resources model. Euro. J. Work Organizational Psychol. 19, 735–759. 10.1080/13594320903223839

[B49] Van den Broeck A. Van Ruysseveldt J. Vanbelle E. De Witte H. (2013). The job demands-resources model: Overview and suggestions for future research, in: Advances in Positive Organizational Psychology, ed A. B. Bakker (Bingley: Emerald Group Publishing Limited). 10.1108/S2046-410X(2013)0000001007

[B50] Van den Broeck A. Vander Elst T. Baillien E. Sercu M. Schouteden M. De Witte H. . (2017). Job demands, job resources,burnout, work engagement, and their relationships: An analysis across sectors. J. Occupat. Environ. Med. 59, 369–376. 10.1097/JOM.0000000000000964 28157768

[B51] Van Wingerden J. Van der Vaart L. (2019). The potential of job demands-resources interventions in organizations, in: Theoretical Approaches to Multi-Cultural Positive Psychological Interventions, eds Van Zyl L. E. Rothmann S. (Cham: Springer). 10.1007/978-3-030-20583-6_5

[B52] Vinod Nair A. McGregor A. Caputi P. (2020). The impact of challenge and hindrance demands on burnout, work Engagement, and presenteeism. A cross-sectional study using the job demands-resources model. J. Occupat. Environ. Med. 62, e392–e397. 10.1097/JOM.0000000000001908 32404829

[B53] Wang Y. Huang J. You X. (2016). Personal resources influence job demands, resources, and burnout: A one-year, three-wave longitudinal study. Soc. Behav. Personal. 44, 247–258. 10.2224/sbp.2016.44.2.247

[B54] Xanthopoulou D. Bakker A. B. Demerouti E. Schaufeli W. B. (2007). The role of personal resources in the job demands-resources model. Int. J. Stress Manag. 14, 121–141. 10.1037/1072-5245.14.2.12133451042

[B55] Xanthopoulou D. Bakker A. B. Demerouti E. Schaufeli W. B. (2009). Reciprocal relationships between job resources, personal resources, and work engagement. J. Vocat. Behav. 74, 235–244. 10.1016/j.jvb.2008.11.003

